# Antioxidant, Antimicrobial Activities and Characterization of Polyphenol-Enriched Extract of Egyptian Celery (*Apium graveolens* L., Apiaceae) Aerial Parts via UPLC/ESI/TOF-MS

**DOI:** 10.3390/molecules27030698

**Published:** 2022-01-21

**Authors:** Ayat M. Emad, Dalia M. Rasheed, Reham F. El-Kased, Dina M. El-Kersh

**Affiliations:** 1 Pharmacognosy Department, Faculty of Pharmacy, October 6 University, Sixth of October City 12511, Egypt; ayatemad@o6u.edu.eg (A.M.E.); Daliarasheed@o6u.edu.eg (D.M.R.); 2 Microbiology & Immunology Department, Faculty of Pharmacy, The British University in Egypt (BUE), Cairo 11837, Egypt; reham.kased@bue.edu.eg; 3 Pharmacognosy Department, Faculty of Pharmacy, The British University in Egypt (BUE), Cairo 11837, Egypt

**Keywords:** celery, *Apium graveolens*, antioxidant, antimicrobial, UPLC/ESI/TOF-MS

## Abstract

Medicinal plant extracts are increasingly considered a major source of innovative medications and healthcare products. This study focused on preparing a polyphenol enriched water extract of Egyptian celery “*Apium graveolens* L., Apiaceae” aerial parts (TAE) in an endeavor to accentuate its antioxidant capacity as well as its antimicrobial activity. (TAE) of celery was partitioned against different organic solvents to yield dichloromethane (DCM), ethyl acetate (EAC), and butanol (BUOH) fractions. (TAE) and the organic fractions thereof besides the remaining mother liquor (ML) were all screened for their antioxidant capacity using various protocols viz. monitoring the reducing amplitudes for ferric ions (FRAP), and radical scavenging potentials of oxygen (ORAC), 2,2’-azino-bis-3-ethylbenzothiazoline-6-sulfonic acid (ABTS), 2,2-diphenyl-1-picrylhydrazyl (DPPH), and metal chelation assays. The examination procedure revealed both (TAE) extract and (DCM) fraction, to pertain the highest antioxidant potentials, where the IC_50_ of the (TAE) using ABTS and metal chelation assays were ca. 34.52 ± 3.25 and 246.6 ± 5.78 µg/mL, respectively. The (DCM) fraction recorded effective results using the FRAP, ORAC, and DPPH assays ca. 233.47 ± 15.14 and 1076 ± 25.73 μM Trolox equivalents/mg sample and an IC_50_ 474.4 ± 19.8 µg/mL, respectively. Additionally, both (TAE) and (DCM) fraction exerted antimicrobial activities recording inhibition zones (mm) (13.4 ± 1.5) and (12.0 ± 1.0) against *Staphylococcus aureus* and (11.0 ± 1.2) and (10.0 ± 1.3) against *Escherichia coli*, respectively, with no anti-fungal activity. Minimum inhibitory concentration (MIC) of (TAE) and (DCM) fraction were 1250 and 2500 µg/mL, respectively. UPLC/ESI/TOF-MS unveiled the chemical profile of both (TAE) and (DCM) fraction to encompass a myriad of active polyphenolic constituents including phenylpropanoids, coumarins, apigenin, luteolin, and chrysoeriol conjugates.

## 1. Introduction

Over the past two decades many edible and medicinal plants have been screened for their antioxidant profiles and indeed have proven to be safe substitutes to some commonly used synthetic antioxidants. Assessment of the antioxidant potential of natural drug extracts is frequently a starting point to validate a variety of other key physiological actions [1]. The correlation between high antioxidant capacities in any plant extract and antidiabetic, anti-inflammatory, antiaging, and anticancer activities has become an established concept in phytotherapy [2]. Additionally, natural antioxidants in the diet proved to boost human immune defenses and prevent oxidative damage to cellular components [3]. However, validation of this capacity requires an array of assessment methods rather than a single assay, to cover the various mechanisms of antioxidant response. Generally, a panel of assays is required to establish antioxidant potential of plant extracts in vitro by various mechanisms viz. radical scavenging, metal chelation, and reducing power assays. Polyphenols have repeatedly proven to be role-playing constituents contributing to strong antioxidant activities in foods and several plant extracts [4,5]. Water extraction of medicinal plants is a relevant approach for pooling of polyphenolic components, as well as generating a safer and more convenient extract/product for certification [5].

Celery (*Apium graveolens* L., Apiaceae) is both a culinary herb and a medicinal plant of wide distribution around the world since antiquity. Powdered celery seeds are used as a spice for their agreeably palatable taste and aromatic odor. In folk medicine it is used as a diuretic, or brewed for relieving hypertension, bronchial asthma, liver and spleen complaints [6]. Chemical examination of celery resulted in recognition and isolation of several flavonoids viz. apigenin, hesperitin, luteolin, and quercitrin conjugates in addition to several well-known phenolic and cinnamic acid derivatives [7]. As a member of the Apiaceae, coumarins of different classes, mainly furanocoumarins, have also been reported in celery [8]. For its rich and diverse contents of phenolic compounds, minerals and vitamin contents, celery has antioxidant, antimicrobial, as well as plethora of other pharmacologically evident actions [9].

The current investigation aimed to validate the antioxidant capacity of the polyphenol enriched water extract (TAE) of celery aerial parts as well as its fractions and the remaining mother liquor upon using different investigation protocols viz. examination of the reducing power (FRAP), scavenging different oxidants (ORAC, ABTS, DPPH), and metal chelation strength. Additionally, the antimicrobial activity of (TAE) and its organic fractions are screened against strains of Gram-positive *Staphylococcus aureus*, Gram-negative *Escherichia coli*, and *Candida albicans* fungi. Finally, the metabolome of the polyphenol-enriched extract (TAE) of celery and its active fractions are assessed using UPLC/ESI/TOF-MS to characterize the metabolic fingerprint promoting their biological activities.

## 2. Results

### 2.1. Evaluation of the Antioxidant Activity In Vitro

In this study the antioxidant capacity of a polyphenol enriched extract (TAE) of celery “*Apium graveolens* L.” aerial parts prepared by aqueous extraction and fractions thereof viz. dichloromethane (DCM), ethyl acetate (EAC), butanol (BUOH), and the remaining mother liquor (ML) prepared as described in material and method Section 4.2, were examined by an array of in vitro assays. A total of 5 assays have been implemented to verify the antioxidant activity of celery (TAE) and fractions thereof through exploring different mechanisms. The examination included probing the reduction capacity of ferric ions to the ferrous state upon incorporation of tested extracts (FRAP), radicle scavenging capacity of three different oxidants viz. ORAC, ABTS, and DPPH, and finally, measurement of the chelation power of the extracts. It has been observed that (TAE) as well as its fractions afforded appreciable antioxidant potentials with the different evaluation protocols as illustrated in (Table 1).

**Figure 1:**
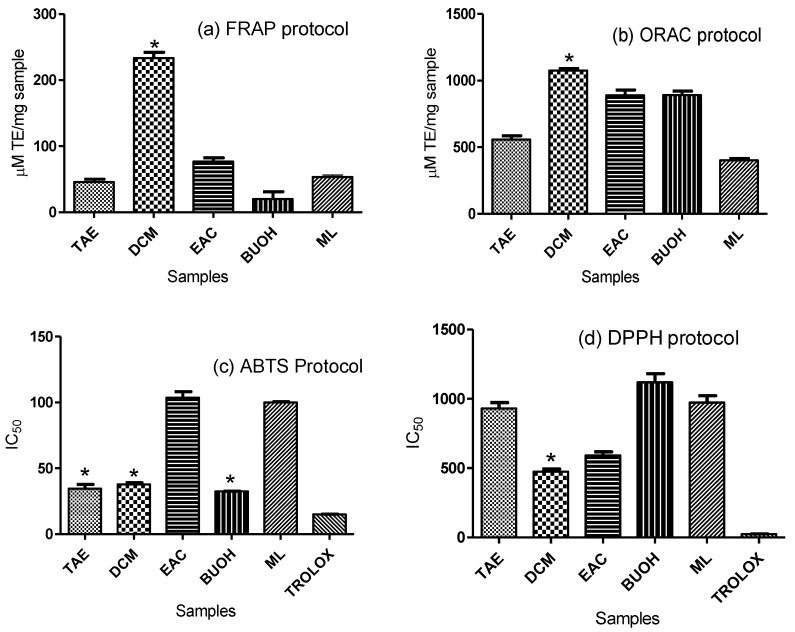

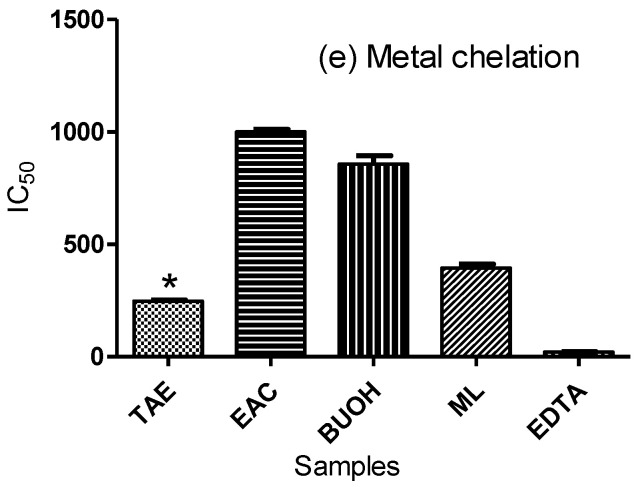
Antioxidant activities of aqueous celery extract and fractions thereof assessed by: (**a**) FRAP, (**b**) ORAC, (**c**) ABTS, (**d**) DPPH, (**e**) metal chelation protocols. TAE: total aqueous extract, DCM: dichloromethane fraction, EAC: ethyl acetate fraction, BUOH: butanol fraction, and ML: remaining mother liquor. In metal chelation protocol (**e**): DCM fraction was not detectable by this assay. *: significant antioxidant activity.

Experimental results also mutually pointed out the (DCM) fraction to pertain the highest antioxidant activity upon using FRAP, ORAC, and DPPH assays. In FRAP and ORAC, the total antioxidant activities have been assessed in Trolox equivalents to record 233.47 ± 15.14 and 1076 ± 45.9 μM TE/mg, respectively. In DPPH protocol, the calculated IC_50_ was 474.4 ± 19.8 µg/mL for (DCM) fraction followed by (EAC) then total aqueous extract (TAE) being 591.4 ± 27.05 and 930.8 ± 42.5 µg/mL, respectively. (TAE) also exhibited effective antioxidant activity upon using other assays as ABTS and metal chelation protocols with recorded IC_50_ values 34.52 ± 3.25 µg/mL and 246.6 ± 5.78 μM eq/mg, respectively. For the record, the observed variability in results among different antioxidant protocols is due to difference in concentrations of tested samples, which is compulsory to work within the linear absorbance range of each assay. The results of all the antioxidant activity investigations are summarized in (Figure 1).

### 2.2. Evaluation of the Antimicrobial Activity

#### 2.2.1. Well Diffusion Assay

The antimicrobial properties of (TAE) extract and its fractions in addition to (ML) at a concentration of 20% *w*/*v* against *Staphylococcus aureus*, *Escherichia coli*, and *Candida albicans* were assessed in this study using the well diffusion assay. The results showed that (TAE) extract and (DCM) fraction suppressed the growth of the tested micro-organisms efficiently with variable potency as explained in Table 2.

The (TAE) extract and (DCM) fraction expressed the maximum zones of inhibition recording (13.4 ± 1.5 mm) and (12.0 ± 1.0 mm) against *Staphylococcus aureus* compared to (11.0 ± 1.2 mm) and (10.0 ± 1.3 mm) against *Escherichia coli*, respectively. (EAC), (BUOH), and (ML) fractions did not show any antibacterial activity against the tested bacteria. Regarding the antifungal activity assessment of all samples, none of the tested extracts showed remarkable zones of inhibition and consequently they had no antifungal activity against *Candida albicans* at selected concentrations.

#### 2.2.2. Determination of Minimum Inhibitory Concentration (MIC)

The lowest concentration that inhibits the growth of bacteria was determined for the (TAE) extract and (DCM) fraction using the minimum inhibitory concentration assay. The results showed that (TAE) extract exhibited more potent antibacterial activity than (DCM) fraction, where total visual bacterial inhibition occurred at concentrations 1250 and 2500 µg/mL, respectively.

### 2.3. UPLC/ESI/TOF-MS Metabolic Profiling of (TAE) of Celery Aerial Parts

**Figure 2:**
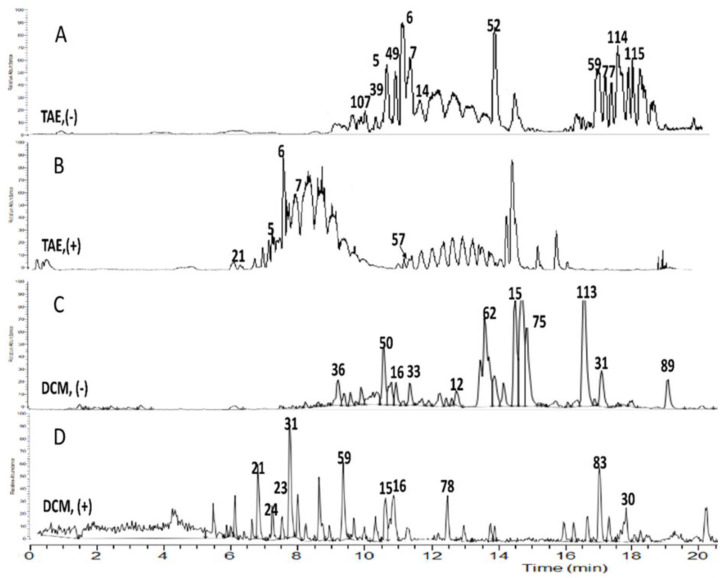
Representative UPLC/TOF-MS base peak chromatograms of celery: (**A**) TAE in negative ESI mode, (**B**) TAE in positive ESI mode, (**C**) DCM in negative ESI mode, and (**D**) DCM in positive ESI mode. Annotated peak numbers follow those listed in Table 3.

Metabolome profiling of (TAE) of celery aerial parts and (DCM) fraction thereof exhibiting promising antioxidant and antibacterial potentials was conducted using reversed phase UPLC/ESI/TOF-MS analysis. Base peak chromatograms representative of (TAE) in both negative and positive ionization modes are given in Figure 2A,B, whereas (DCM) chromatograms are depicted in Figure 2C,D, respectively.

The analytical setup adopted herein allowed for the simultaneous identification of 115 plant metabolites belonging to chemically variant classes within ca. 20 min that are listed in Table 3.

**Figure 3:**
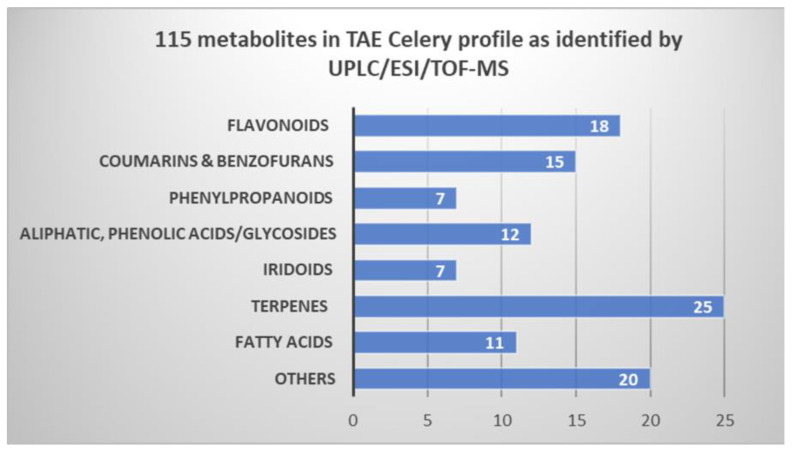
Phytochemical classes of the 115 metabolites tentatively identified in (TAE) extract using UPLC/ESI/TOF-MS.

The following section discusses the identification of major secondary metabolites of biological merit and summarized in Figure 3.

#### 2.3.1. Flavonoids

Celery (TAE) profile revealed abundancy in the flavonoid composition where 18 metabolites mainly flavones including apigenin, chrysoeriol, and luteolin aglycones and conjugates thereof as reported previously, in addition to one flavanol which was tentatively assigned as noidesol A (C-hexosyl methoxyflavanol) [10,11,12]. Apigenin conjugates dominated the celery (TAE) profile as it was assigned in half of the flavonoid configuration (9 metabolites) which could be attributed to the aqueous extraction method adopted. Chrysoeriol (3’-O-methyl derivative of luteolin) and its conjugates were assigned in 6 of the identified flavonoids based on the fragment ion *m*/*z* 299.05548 [M-H]^−^ with the predicted chemical formula [C_16_H_11_O_6_]^−^. Luteolin-*O*-hexosyl pentoside (*m*/*z* 579.13342, peak **5**), apiin (*m*/*z* 563.13843, peak **6**), and chrysoeriol-*O*-hexosyl pentoside (*m*/*z* 593.14832, peak **7**) were major peaks in negative mode (Figure 2), revealing neutral losses of (162 Da) and (132 Da) of the molecular ion, indicative of *O*-linked hexoside and pentose residues, respectively [13] (Figures S1–S3).

For example, peaks **3** and **4** (*m*/*z* 727.20697 [C_32_H_39_O_19_]^+^ and *m*/*z* 757.21661 [C_33_H_41_O_20_]^+^) at t_R_ = 10.12 and 10.35 min., respectively, demonstrated similar fragment patterns and generating daughter ions at *m*/*z* 595.16425, 433.11163, 271.05960, and *m*/*z* 625.17542, 463.12268, 301.07040 Da, respectively, indicating *O*-linked pentose [M+1-132]^+^, *O*-linked pentose-hexose [M+1-132-162]^+^, and *O*-linked pentose-dihexose sugar moieties [M+1-132-162-162]^+^, respectively (Figures S4 and S5). Consequently, metabolite # 3 was annotated as apigenin-*O*-dihexosyl pentoside, whereas metabolite # 4 was annotated chrysoeriol-*O*-dihexosyl pentoside as reported in literature [14]. Similarly, peaks **9** and **10** (*m*/*z* 741.20148 Da [C_36_H_37_O_17_]^+^ and *m*/*z* 771.21106 Da [C_37_H_39_O_18_]^+^) at t_R_ = 12.15 and 12.37, respectively, generated daughter ions representative for losses of [M+1-162]^+^, [M+1-162-146]^+^, [M+1-162-146-162]^+^, respectively, and supported an *O*-linked three sugar structure. Metabolites 9 and 10 were assigned to be apigenin-*O*-dihexosyl deoxy hexoside and chrysoeriol-*O*-dihexosyl deoxy hexoside, respectively (Figures S6 and S7).

#### 2.3.2. Coumarins and Benzofurans

Coumarins are pervading and important biologically active secondary metabolites in the family Apiaceae as well as other plant families viz. Asteraceae, Moraceae, and Rutaceae [15]. Apiaceae harbors diverse chemical structures of coumarins which are regarded as chemotaxonomic markers [15]. The (TAE) profile of celery encompassed 15 coumarin compound of varying configurations viz. furanocoumarins (isopimpinellin and marmesin), furanochromone (khellin), hydroxycoumarins (methylumbelliferone and isofraxidin), coumarinolignoid (cleomiscosin A), and coumarin glycosides (aesculin).

A major coumarin metabolite was observed at t_R_= 14.69 min. Peak 32, at *m*/*z* 247.06464 Da [C_13_H_11_O_5_]^+^ with a daughter base peak at *m*/*z* 232.03647 Da and predicted chemical formula [C_12_H_8_O_5_]^+^ implying the loss of methyl group [M-CH_3_]^+^ was annotated as isopimpinellin (Figure S8) as reported by [16]. Similarly, a less abundant peak **21** at t_R_ = 6.78 min. with molecular ion *m*/*z* 223.0602 Da, [C_11_H_11_O_5_]^+^ showed a base peak at *m*/*z* 208.03674 Da, [C_10_H_8_O_5_]^+^ for the loss of methyl group [M-CH_3_]^+^, and peak **21** was assigned to isofraxidin (Figure S9). Its *O*-hexoside derivative was assigned to peak **22** at t_R_ = 7.41 min. with molecular ion *m*/*z* 383.09430 Da [C_17_H_19_O_10_]^−^ which generated a base peak at *m*/*z* 221.04509 Da [C_11_H_9_O_5_]^−^ designating the loss of hexosyl moiety (Figure S10).

Khellin is a major constituent of *Ammi visnaga,* and was identified in other species in family Apiaceae [17] and was assigned to the molecular ion *m*/*z* 261.07565 Da [C_14_H_13_O_5_]^+^ (peak **28** at t_R_ = 13.53 min.) which showed a base peak at *m*/*z* 246.05215 Da [C_13_H_10_O_5_]^+^ referring to a loss of methyl group (Figure S11). Respectively, peak **25** (t_R_ = 10.66 min.) was annotated as isopimpinellin-*O*-hexoside, with the molecular ion at *m*/*z* 409.11224 [C_19_H_21_O_10_]^+^ and base peak at *m*/*z* 247.06020 Da [C_13_H_11_O_5_]^+^ [M-162]^+^ referring to isopimpinellin after loss of *O*-hexosyl moiety (Figure S12).

#### 2.3.3. Phenylpropanoids

Seven phenylpropanoids were identified in (TAE) of celery mainly as quinic acid conjugates as a consequent of extraction with water. Quinic acid fragments were observed in peaks **34** and **36** as base peaks at *m*/*z* 191.05551 Da with the predicted chemical formula [C_7_H_11_O_6_]^−^, in addition to fragments at *m*/*z* 179.03462 [C_9_H_7_O_4_]^−^ and *m*/*z* 163.03981 Da [C_9_H_7_O_3_]^−^ corresponding to caffeoyl and coumaroyl moieties, respectively (Figures S13 and S14). On the other hand, peak **37** at t_R_ = 9.87 min. with molecular ion *m*/*z* 369.11801 Da, [C_17_H_21_O_9_]^+^ showed a base peak at *m*/*z* 177.05449 Da [C_10_H_9_O_3_]^+^ representing the loss of quinic moiety [M+1-191]^+^ (Figure S15). Phenylpropanoids have been already reported in family Apiaceae, and their antimicrobial and antioxidant activities are well established [18].

#### 2.3.4. Terpenes

Plenty of low intensity peaks were ascribed to triterpenes and steroids (25 metabolite) in (TAE) profile of celery, which could be a result of aqueous extraction. Identified metabolites include 6 triterpenes (peaks # 70, 71, 73, 74, 83, and 84), 4 sesquiterpenes (peaks # 60, 61, 62, and 81), and a steroidal terpene (peaks # 68). The terpene glycosides citroside A (peak **63**), was detected at t_R_ = 9.31 min. at *m*/*z* 385.18555 Da with predicted chemical formula [C_19_H_29_O_8_]^−^, and the daughter fragment ion at *m*/*z* 205.1230 Da [C_13_H_17_O_2_]^−^ implies the neutral losses of a hexose and water portions [M+1-162-18]^+^ (Figure S16). Auraptene (peak **75** at t_R_ = 16.72 min. at *m*/*z* 297.15207 Da [C_19_H_21_O_3_]^−^) is a bioactive monoterpene coumarin ether which has been reported in celery to possess strong antioxidant and hepatoprotective activities [19] (Figure S17). A unique terpenoid ester, tschimganin, reported previously in family Apiaceae [20], was identified in (TAE) (peak **78** at t_R_ = 17.79 min. at *m*/*z* 305.17484 Da [C_18_H_25_O_4_]^+^), showing a predominant fragments at *m*/*z* 273.14832 Da [C_17_H_21_O_3_]^+^ referring to the losses of hydroxyl and methyl moieties [M+1-17-15]^+^, respectively (Figure S18).

## 3. Discussion

Celery (TAE) extract and its (DCM) fraction, pertained the most significant antioxidant and antimicrobials potentials as described in previous Section 2.1 and Section 2.2. Hence, it was crucial to correlate the biological results with the chemical profiling of celery using UPLC/ESI/TOF-MS to understand the influence of these natural secondary metabolites as well as their previously reported mechanisms of action if present.

### 3.1. Evaluation of Antioxidant Activity in Correlation to UPLC/ESI/TOF-MS Metabolite Profiling

The polyphenol-enriched extract of celery aerial parts and its fractions exerted effective antioxidant activity upon using different antioxidant protocols in agreement with several in vitro and in vivo studies of alcoholic extract and juice of celery herb and seeds [7,21,22,23]. In the current study, results of thorough antioxidant examinations acknowledged both celery (TAE) and (DCM) as fractions with high antioxidant potential, hence characterizing their metabolome profile was performed using UPLC/ESI/TOF-MS. Under the analytical setup procedure described in Section 4.5.3, a total of 115 metabolites were separated and identified. The phytochemical constitution of celery (TAE) entailed a total of 18 flavone conjugates, 15 coumarin and benzofuran derivatives, 7 phenylpropanoids, 12 aliphatic and phenolic acids, and 25 terpenoid compounds as well as other metabolites as illustrated in Table 3 and Figure 2A,B in Section 2.3.

The flavonoid fingerprint of celery (TAE) was composed almost entirely of apigenin, luteolin, and chrysoeriol conjugates which were all reported to possess varying antioxidant features [24]. Owing to the aqueous extraction, the glycosidic configuration predominated the flavonoid fingerprint which imposes an impact on the overall antioxidant activity as research studies have conducted that glycoside are generally stronger antioxidants than their respective aglycones [25]. Apiin (peak 6, t_R_ = 11.40), detected with high abundance in (TAE) of celery aerial parts is the apigenin flavone diglycoside marker of Apiaceae members, established antioxidant properties both in vitro and in vivo models (22). On the other hand, the (DCM) fraction seemed to pertain several coumarins which were assigned to the peaks of higher intensity observed in its UPLC/MS analytical chromatogram Figure 2C,D. Coumarins are characteristic heterocyclic compounds of superb thermal and photo stabilities and have been associated with several beneficial effects on human health. Coumarins of family Apiaceae and reported herein in the metabolite profiles of tested celery fractions, (TAE) and (DCM), have reported antioxidant activities [26]. Phenylpropanoids with their chief representative, caffeic acid have been recognized as potent antioxidants compounds in numerous in vitro and in vivo assays [27]. Phenolic acids and phenolic glycosides were also highly abundant metabolites in celery fractions owing to aqueous extraction (Figure 2A–D). The assigned phenolic acids in celery (TAE) and/or (DCM) extracts viz. elenolic and azelaic are recognized antioxidants, the former attributes for the natural antioxidant activity of extra virgin olive oil, while the latter is extensively employed in natural cosmetic preparations [28,29]. Based on these findings, it can be deduced that celery’s antioxidant effect is evidently related to its polyphenol enriched chemical profile.

### 3.2. Evaluation of Antimicrobial Activity in Correlation to UPLC/ESI/TOF-MS Metabolite Profiling

*Apium graveolens* extracts were screened for their antimicrobial effects against *Staphylococcus aureus* representing Gram-positive bacteria, *Escherichia coli* representing Gram-negative bacteria, and *Candida albicans* representing fungal strain. The antimicrobial effects were tested using well diffusion assay and MIC. Out of the 5 extracts tested, only (TAE) extract and (DCM) fractions were demonstrated to possess inhibition against Gram-positive and -negative strains ranging between 9–15 mm, while they did not give anti-fungal activity. The values of MIC of *Apium graveolens* (TAE) and (DCM) were 1250 and 2500 µg/mL, respectively; showing that (TAE) was more potent. A previous study on extract of celery recorded effective inhibitory action on both *Staphylococcus aureus* and *S. epidermidis* which proves the efficacy of celery as an antibacterial agent [30], whereas celery also proved its efficacy on cytokeratin and other healing factors in wounds of infected skin with methicillin resistant *S. aureus* strains as well [31]. Many studies have demonstrated the antimicrobial potency of plant extracts and their bioactive components—solely or in groups with other components—flavonoids being the highest phytochemical showing antibacterial [32] as well as antifungal activities [33]. The suggested mechanisms of antibacterial action are microbial plasma membrane degradation, DNA topoisomerase inhibition, and inhibition of microbial energy metabolism [32,34].

The results demonstrated in this study show that *Apium graveolens* (TAE) and (DCM) extracts inhibited bacterial growth in the test cultures. However, *Candida albicans* was resistant to all extracts at the applied concentrations. It could be observed that higher concentrations of the extracts possesed more bacterial inhibition. The slight differences in sensitivity to the effect of different extracts between the Gram-negative and Gram-positive bacteria shown in this study are supported by other studies in literature [35,36]. The mechanism which explains the weak susciptibility of Gram-negative bacteria is not exactly known. However, it may be attributed to the strong hydrophobic outer membrane of Gram-negative bacteria which acts as a strong permeability barrier [37]. The antibacterial effect of *Apium graveolens* (TAE) compared to (DCM) could be related to the high content of phenolics exmplified in flavonoids [38], as well as to some extent to its volatiles content, mainly the aromatic components and *D*-limonene, which could affect the polarity and consequently the degree of bacterial inhibition [39].

## 4. Material and Method

### 4.1. Chemicals, Reagents, and Microbial Strains

HPLC grade solvents—acetonitrile and methanol—were purchased from Thermo-Fisher Scientific Co. (Waltham, MA, USA). Mobile phase solvents viz. ammonium hydroxide and formic acid 98% were purchased from Sigma–Aldrich Co. (St. Louis, MO, USA). All other chemicals (TPTZ “tripyridyl-1- s-triazine”, AAPH “2,2′-azobis (2-amidinopropane) dihydrochloride”, ABTS “2, 2′-azino-bis (3- ethylbenzothiazoline-6-sulfonic acid), ferrous sulphate, DPPH (2,2-diphenyl-1-picryl-hydrazyl-hydrate), and solvents (dichloromethane, ethyl acetate, and butanol) were of analytical grades and bought from Sigma Aldrich Company.

Microbial strains tested represented Gram-positive bacteria [*Staphylococcus aureus* ATCC 25923], Gram-negative bacteria [*Escherichia coli* ATCC 25922], as well as one pathogenic fungus [*Candida albicans* RCMB 005003 (1) ATCC 10231].

### 4.2. Plant Material and Sample Preparation

Aerial parts of celery “*Apium graveolens* L.” were gathered in Spring from Fayoum governorate just before flowering, authenticated by Prof. Dr. Wafaa M. Amer, Cairo University Herbarium, Faculty of Science. Celery aerial parts were carefully dried in the shade then powdered. The polyphenol-enriched extract was prepared by macerating 750 g of the dried plant material in distilled water overnight. Total aqueous extract (TAE) was concentrated on rotary evaporator (R-210 evaporator, Büchi, Switzerland) till formation of a concentrated, viscous extract. Seventy-five ml of the concentrated extract was completely dried by lyophilization (LGJ-10, Mingyi, China, Dongguan, China) to yield 50 g of (TAE) for biological and UPLC/ESI/TOF-MS examinations. The remaining viscous extract was partitioned between organic solvents with increasing polarity viz. dichloromethane (DCM), ethyl acetate (EAC), and butanol (BUOH). Each organic fraction was evaporated to dryness to yield 1.58, 2.54, and 21.41 g, respectively, and the remaining mother liquor (ML) was also concentrated under vacuum and freeze dried (23.78 g).

Serial dilutions of the 5 samples—total aqueous extract (TAE) and fractions thereof viz. (DCM), (EAC), (BUOH), and (ML)—were prepared in concentrations of µg/mL for carrying out the array of antioxidant examination procedures as will be described within each protocol.

### 4.3. Evaluation of the Antioxidant Activity

All results assessed in all antioxidant protocols using the microplate reader FluoStar Omega (BMG LABTECK) and expressed as averages ± SD of triplicate measurements. The antioxidant capacity for the tested samples was expressed as μM Trolox equivalent “TE”/mg of each sample following the equation y = 0.0019x + 0.0874 (R^2^ = 0.9985) or y = 377.7x + 29731 (R^2^ = 0.9972) in FRAP or ORAC protocol, respectively.

IC_50_ values were calculated from free radical scavenging activity (% inhibition) versus log concentration of sample curve (µg/mL). IC_50_ values of tested samples in ABTS, DPPH, and metal chelation protocols were calculated using Graph pad Prism 6 with the data represented as mean ± SD of triplicate measurements using the equation:

Percentage inhibition = ((Blank average absorbance -Test average absorbance)/(Blank average absorbance)) × 100

The antioxidant capacity results of different samples were compared using ANOVA test followed by Tukey’s post hoc test, whereas significant *p*-value was (*p* < 0.05) using Graph pad prism 6.

#### 4.3.1. FRAP Assay “Trolox Equivalent”

Antioxidant activity in vitro using ferric reducing antioxidant power “FRAP” was carried out on the 5 samples under examination: viz. (TAE), (DCM), (EAC), (BUOH), and (ML). FRAP antioxidant protocol was assessed as per the method described briefly by Benzie, I.F. and Strain, J.J. [40] with minor modifications to suit microplate recording. A freshly prepared TPTZ “tripyridyl-1-s-triazine” reagent (190 µL) (300 mM of buffer acetate PH 3.6 added to 10 mM of TPTZ dissolved in 40 mM of hydrochloric acid and 20 mM of ferric chloride mixed in ratios of 10:1:1 *v*/*v*/*v*; respectively) is added to each sample (10 µL) separately (concentration =1 µg/mL) in a 96-well plate. Triplicate reaction mixtures were incubated for half an hour in the dark at 37 °C, and the blue color distinctive of ferrous ion generation was measured at λ 593 nm. Trolox standard curve of prepared in 25, 50, 100, 200, 400, 600, and 800 µM serial dilutions.

#### 4.3.2. ORAC Assay “Trolox Equivalent”

The ORAC “Oxygen Radical Absorbance Capacity” was carried out in vitro for the 5 test samples compared to Trolox standard. The method was conducted as per Liang et al. [41] with some modifications: 10-μL of each test prepared sample “1 mg/mL” was incubated separately with 30 μL of fluoresceine (100 nM) for 10 min at room temperature. Fluorescence measurement was conducted for 3 cycles (485 EX, 520 EM, nm-cycle time, 90 s) for measurements of the background followed by 70 μL of freshly prepared 2,2′-azobis (2-amidinopropane) dihydrochloride (AAPH) and 300 mM was added directly to each well “96-well plate, *n* = 3”. Fluorescence measurements (485 EX, 520 EM, nm) was continued for an hour at (40 cycles, each 90 s). Trolox standard curve was prepared using serial concentrations of 50, 100, 200, 400, 500, 800, and 1000 μM in 1 mM of methanol.

#### 4.3.3. ABTS Radical Scavenging Assay

The antioxidant ABTS “2, 2′-azino-bis (3-ethylbenzothiazoline-6-sulfonic acid)” assay was conducted as in the method described by Arnao et al. [42] with minor modifications to suit microplate recording. ABTS reagent (192 mg) was dissolved in distilled H_2_O and adjusted to 50 mL. Then, 1 mL of the prepared solution was added to 17 µL (140 mM potassium persulphate). The mixture was left in the dark at room temperature for 24 h, and then 1 mL of the reaction mixture was completed to 50 mL with methanol to obtain a final ABTS reagent for the assay. The freshly prepared ABTS (190 µL) was mixed with each sample of volume of 10 µL in the 96-well plate (*n* = 6). The reaction was incubated in the dark for half an hour. The 5 test samples were prepared separately in serial dilutions of 5, 20, 40, 60, 80, and 100 µg/mL, whereas the Trolox standard serial concentrations were 1.64, 3.28, 6.57, 13.15, 26.31, and 39.47 µM. Quenching of ABTS coloration was measured at λ 734 nm.

#### 4.3.4. DPPH Radical Scavenging Assay

The antioxidant IC_50_ of the 5 test samples were further investigated using DPPH free radical protocol following the procedure described by Boly et al. [43]. DPPH (100 μL) in concentration (0.1% in methanol) was added to each sample (100 μL) in a 96-well plate (*n* = 6). The reaction was incubated at 37 °C for 30 min. in the dark. The reduction in color intensity of the DPPH was measured at λ 540 nm. The samples’ serial dilution was prepared each from stock solution in methanol “1 mg/mL” in dilutions of 100–1000 µg/mL, whereas Trolox (100 µM in methanol) serial dilutions were 10, 15, 20, 30, 40, and 50 µM.

#### 4.3.5. Metal Chelation Assay

Metal chelation assay was conducted as previously described by Santos et al. [44]. A 20 µL of freshly prepared ferrous sulphate (0.3 mM) was mixed with 50 µL of each of the 5 test samples separately in 96 well plate (*n* = 3). A 30 µL of ferrozine (0.8 mM) was added to each well. Serial dilutions of 200, 400, 600, 800, and 1000 µg/mL for each sample was prepared as well as EDTA serial dilutions of 2.5, 5, 10, 20, and 25 µM from (0.05 mM) stock solution. Reaction mixtures were left for 10 min in dark at 37 °C. Quenching of the generated color intensity was assessed at λ 562 nm.

### 4.4. Evaluation of the Antimicrobial Activity

#### 4.4.1. Well Diffusion Assay

Both *Staphylococcus aureus* and *Escherichia coli* were pre-cultured in Mueller Hinton broth (MHB) overnight in a rotary shaker at 37 °C. Afterwards, each strain was adjusted at a concentration of 10^8^ cells/mL using 0.5 of McFarland standard [45]. Sabouraud dextrose broth (SDB) was used to prepare fungal inoculum after the 48 h culture of fungal isolates [46]. The spore density of *Candida albicans* was adjusted to a final concentration of 10^6^ spores/mL using a spectrophotometer (A_595_ nm). Screening of the antibacterial and antifungal activities of different samples was done using Agar well diffusion method [47]. Using a sterile Petri dish, one ml of fresh bacterial or fungi culture was pipetted in the center. Molten cooled Muller Hinton agar (MHA) for bacteria and Sabouraud dextrose agar (SDA) for fungi was then poured into the Petri dish containing the inoculum then mixed well. After agar solidification, a sterile cork borer was used to make wells into agar dishes containing inoculums (6 mm in diameter); each plate contained 6 wells. This was followed by addition of 100 μL of each extract (20% *w*/*v*) to respective wells in each dish. Then, the plates were refrigerated for 30 min to allow the diffusion of the extracts into the agar. Afterwards, the plates were incubated at 37 °C for 18 h. Antimicrobial activity was detected by measuring the zone of inhibition (including the wells diameter) appeared after the incubation period. Gentamycin (4 μg/mL) and ketoconazole (100 μg/mL) were used as positive controls for bacteria and fungi, respectively, while 10% DMSO was used as a negative control.

#### 4.4.2. Minimum Inhibitory Concentration (MIC)

Broth dilution method was used for MIC determination using 96-well microplates. Serial dilutions (100 μL) of the tested extracts were used covering the concentration range of 62.5 to 4000 μg/mL in Mueller–Hinton broth (Sigma–Aldrich, St. Louis, MO, USA). Bacterial inocula (100 μL) were prepared from 18-h broth culture (containing 10^5^ cfu/mL), then poured into the wells. This was followed by incubation at 37 °C for 24 h. Three triplicates were made.

### 4.5. Metabolite Profiling of Celery Extract Using UPLC/ESI/TOF-MS

#### 4.5.1. Celery Extract Preparation

Examination was carried out in accordance to the procedure described by Mohammed et al. [48]. Stock solutions of the active celery extracts (TAE) and (DCM) were prepared using 50 mg of each extract, dissolved separately in 1 ml of a reconstitution solvent composed of (water:methanol:acetonitrile, 50:25:25 *V*/*V*). Mixtures were vortexed for 2 min, dissolved completely by ultra-sonication for 10 min then centrifuged at 10,000 rpm for 5 min. Fifty µL of stock solutions were diluted to 1000 µL using the reconstitution solvent to obtain a final concentration of 2.5 µg/µL. Ten µL of each prepared sample was injected in both positive and negative ionization modes. The LC-MS analysis procedure was also applied for blank, quality control samples, and internal standard used for validation of the experiment setup.

#### 4.5.2. Instrument and Spectral Acquisition

The analysis was performed using an ExionLC analytical UHPLC system (SCIEX, Framingham, USA) equipped with a column (Waters, Xbridge C-18, 50 × 2.1 mm, 3 µm particle size) operated at 40 °C, and precolumn (In-Line filter discs, Phenomenex, 0.5 µm × 3.0 mm). There were 3 mobile phases used: mobile phase (A) 5 mM of ammonium formate buffer pH 3 in 1% methanol, mobile phase (B) 5 mM of ammonium formate buffer pH 8 in 1% methanol, and mobile phase (C) 100% acetonitrile. Solvents (B) and (C) were used for the negative ion mode, while solvents (A) and (C) were used for the positive ion mode. Gradient elution was performed at a flow rate of 0.3 mL/min at 40 °C, where from 0 to 1 min, isocratic (90% (A or B), 10% (C)), from 21 to 25 min, isocratic 10% (A) or (B), to 90% (C). From 25.01 to 28 min, elution was isocratic (90% (A or B), 10% (C)), until equilibrium.

Mass spectrometry was performed using a Triple TOF 5600+ system, operating in the ESI mode and having a Duo-Spray source (SCIEX, Concord, ON, Canada). The sprayer and declustering voltages were set at 4500 and 80 eV in the positive ESI mode, and −4500 and −80 V in the negative ESI mode. Source temperature = 600 °C, collision energy = 35 V/−35 V in positive/negative modes, CE spreading 20 V, and the ion tolerance for 10 ppm were used. IDA protocol (information-dependent acquisition) was used for TripleTOF5600+ operation. MS/MS data were generated using Analyst-TF 1.7.1 software. Full-scan MS and MS/MS information of high-resolution survey spectra from 50 to 1100 *m*/*z* were obtained.

#### 4.5.3. UPLC/ESI/TOF-MS Data Processing

Peakview 2.2 software (SCIEX, Framingham, MA, USA) was used for data processing and to record the retention time and masses of the detected molecules (Tsugawa, Cajka et al. 2015). The detected masses ranged from 50 to 1000 Da. Metabolites were assigned tentatively by matching mass spectral data of the identified metabolites in both ionization modes with reported data provided in online libraries and databases (Human Metabolome Database and Pubchem), alongside retention times and fragmentation patterns comparison to reference standards whenever possible.

## 5. Conclusions

To cope with the growing demands of pharmaceutical industry for biologically active natural extracts it is crucial to extract medicinal plants in a proper manner. Medicinal plant extracts with evidently strong antioxidant activity will by far pertain to accentuated pharmacological actions. In this study, the polyphenol enriched extract prepared by water extraction of celery aerial parts exhibited evident antioxidant capacity in response to an array of different assays as well as promising antibacterial activity. UPLC/ESI/TOF-MS profiling verified the accumulation of flavonoids, phenylpropanoids, and other active secondary metabolites in this extract and recommends its incorporation in dietary supplements. The research workflow adopted herein can be applied to other edible and/or medicinal plants for probing their antioxidant and antimicrobial potentials as a preliminary step for exploring their pharmacological effectiveness.

## Figures and Tables

**Table 1:** Antioxidant activity of the total aqueous extract of celery aerial parts (TAE) and fractions thereof; (DCM), (EAC), (BUOH), and (ML) by various protocols.

Antioxidant Assay	TAE (Average ± SD)	DCM (Average ± SD)	EAC (Average ± SD)	BUOH (Average ± SD)	ML (Average ± SD)
FRAP μM TE/mg sample	45.57 ± 4.46	233.47 ± 15.14	76.80 ± 9.68	19.75 ± 19.76	53.47 ± 5.02
ORAC μM TE/mg sample	558.74 ± 45.90	1076 ± 25.73	888.83 ± 70.50	890.18 ± 53	402.02 ± 23.18
ABTS “IC_50_” µg/ml	34.52 ± 3.25	37.78 ± 1.24	103.7 ± 4.47	32.56 ± 0.09	100.0 ± 0.58
DPPH “IC_50_” µg/ml	930.8 ± 42.50	474.4 ± 19.80	591.4 ± 27.05	1119 ± 62.30	973.4 ± 50.21
Metal chelation “IC_50_” µg/ml	246.6 ± 5.78	nd	1000 ± 10.40	856.7 ± 37.13	394.1 ± 17.81

nd: not detected under experimental conditions.

**Table 2:** Antimicrobial activity of total aqueous extract of celery aerial parts (TAE) and fractions thereof; (DCM), (EAC), (BUOH), and (ML) against three microorganisms.

	TAE Average (mm) ± SD	DCM Average (mm) ± SD	EAC Average (mm) ± SD	BUOH Average (mm) ± SD	ML Average (mm) ± SD
Zones of inhibition					
*Staphylococcus aureus*	13.4 ± 1.5	12.0 ±1.0	nd	nd	nd
*Escherichia coli*	11.0 ± 1.2	10.0 ± 1.3	nd	nd	nd
*Candida albicans*	nd	nd	nd	nd	nd

nd: no zone of inhibition was detected.

**Table 3:** Metabolites tentatively identified in (TAE) and (DCM) of celery aerial parts (*Apium graveolens* L.) via UPLC/ESI/TOF-MS in negative and positive ESI ionization modes.

Peak #	Rt (min.)	Metabolite Name	Mol. Ion *m*/*z* (−)/(+)	Elemental Composition	Δ Mass (ppm)	MS^2^ Ions *m*/*z* (−)/(+)	DCM Fraction
*Flavonoids*							
1	8.00	Noidesol A	479.11841	C_22_H_23_O_12_^−^	0.02	451.12241, 317.06561	
2	9.53	Apigenin-*C*-dihexoside	593.14972/ 595.16516	C_27_H_29_O_15_^−^/ C_27_H_31_O_15_^+^	0.64/ −0.98	473.10767, 353.06583/577.15472, 457.11240, 325.07031	
3	10.12	Apigenin-*O*-dihexosyl pentoside	727.20697	C_32_H_39_O_19_^+^	−1.42	595.16425,433.11163, 271.05960	
4	10.35	Chrysoeriol-*O*-dihexosyl pentoside	757.21661	C_33_H_41_O_20_^+^	−1.36	625.17542,463.12268, 301.07040, 286.04706	
5	10.85/10.95	Luteolin-*O*-hexosyl pentoside *	579.13342/ 581.14941	C_26_H_27_O_15_^−^/ C_26_H_29_O_15_^+^	1.77/ −1.18	447.09232,285.03989/449.10730, 287.05487	
6	11.40/14.02	Apiin *	563.13843/ 565.15326	C_26_H_27_O_14_^−^/ C_26_H_29_O_14_^+^	1.96/ −3.401	431.09698,269.04495/433.11264, 271.06009	
7	11.50/12.13	Chrysoeriol-*O*-hexosyl pentoside *	593.14832/ 595.16650	C_27_H_29_O_15_^−^/ C_27_H_31_O_15_^+^	2.96/ 1.26	461.10800,299.05548, 284.03226/463.12268, 301.07031	
8	12.10	Chrysoeriol malonyl-*O*- hexosyl pentoside	681.16431	C_30_H_33_O_18_^+^	0.12	549.12354, 301.07050	
9	12.15	apigenin-*O*-dihexosyl deoxy hexoside	739.1839/ 741.20148	C_36_H_35_O_17_^−^/ C_36_H_37_O_17_^+^	−1.41	545.12793, 269.04468/579.14844, 433.11194, 271.05972	
10	12.37	Chrysoeriol-*O*-hexoside deoxyhexoside hexoside	771.21106	C_37_H_39_O_18_^+^	−1.53	463.12256, 301.07043	
11	12.56	Apigenin-*O*-acyl hexosyl pentoside	607.16412	C_28_H_31_O_15_^+^	−2.68	475.12262, 271.05981	
12	12.68	Luteolin	287.05487	C_15_H_11_O_6_^+^	−0.50	270.14902, 258.05222	+
13	12.75	Chrysoeriol acyl-*O*-hexosyl pentoside	637.17535	C_29_H_33_O_16_^+^	−1.51	505.13290, 301.07010	
14	13.31	Apigenin diacyl-*O*-deoxyhexosyl pentoside *	633.17981	C_30_H_33_O_15_^+^	−0.86	501.13834, 271.05984	
15	13.39/13.43	Apigenin	269.04465/271.05981	C_15_H_9_O_5_^−^/ C_15_H_11_O_5_^+^	0.74/ −1.07	225.05508,149.02399/214.09019, 153.01828	+
16	13.73	Chrysoeriol	301.07059	C_16_H_13_O_6_^+^	−0.25	286.04684, 181.04985	+
17	14.03	Apigenin-*O*-hexosyl dimethyl caffoeyl-*C*-pentoside	761.2627	C_37_H_45_O_17_^+^	7.79/ 4.52	629.22205, 271.06000	
18	19.93	Apigenin-*O*-acyl (trimethyl ether) methoxy galloyl quinoyl (acyl pentosyl) pentoside	803.38306	C_42_H_59_O_15_^+^	−2.23	671.34082, 271.05969	
*Coumarins and Benzofurans*							
19	0.95	Methylumbelliferyl acetate	217.048	C_12_H_9_O_4_^−^	−6.94	181.07140	
20	2.30	Aesculin	339.07104	C_15_H_15_O_9_^−^	−0.05	177.01891	
21	6.78	Isofraxidin *	223.0602	C_11_H_11_O_5_^+^	0.45	208.03674, 163.03891	
22	7.41	Isofraxidin-*O*-hexoside	383.0943	C_17_H_19_O_10_^−^	−3.76	221.04509, 193.05090	
23	9.09	Umbellifolide	265.14282	C_15_H_21_O_4_^+^	−2.32	247.13225,203.10645, 175.07507	
24	9.90	Dihydroxybiscoumarin	323.05542	C_18_H_11_O_6_^+^	1.26	295.05978, 267.06494, 149.02306	
25	10.66	Isopimpinellin-*O*-hexoside	409.11224	C_19_H_21_O_10_^+^	−1.67	247.06020	
26	12.92	Cleomiscosin A	387.10712	C_20_H_19_O_8_^+^	−0.84	369.09616, 337.07004, 161.05956	
27	13.05	Licocoumarone	341.1376	C_20_H_21_O_5_^+^	−2.20	323.12717, 271.09604, 137.05949	
28	13.53	Khellin	261.07565	C_14_H_13_O_5_^+^	−0.38	246.05215	
29	13.54	Visnagin/Desmethoxykhellin	231.065	C_13_H_11_O_4_^+^	−0.80	216.04149, 175.03882	+
30	13.60	Encecalin/methyleupatoriochromene	233.11469	C_14_H_17_O_3_^+^	−10.86	217.04517	+
31	14.35	Senkyunolide F	205.08675	C_12_H_13_O_3_^−^	−4.04	161.09692, 148.01640, 132.05791	+
32	14.70	Isopimpinellin (Dimethoxypsoralen)	247.06464	C_13_H_11_O_5_^+^	−1.34	232.03647	+
33	16.48	Marmesin	245.08145	C_14_H_13_O_4_^−^	−2.51	203.03471, 161.02415	+
*Phenylpropanoids*							
34	4.17/3.70	Caffeoylquinic acid	353.08698/355.10165	C_16_H_17_O_9_^−^/C_16_H_19_O_9_^+^	0.77/ −1.99	233.04478, 191.05551, 179.03462/337.09155, 289.07062, 193.04955, 163.03885	
35	5.09	Caffeic acid	179.03452	C_9_H_7_O_4_^−^	−3.55	135.04529	+
36	8.73/8.86	Coumaroylquinic acid	337.09204/339.10663	C_16_H_17_O_8_^−^/ C_16_H_19_O_8_^+^	0.73/ −2.40	191.05577, 163.03981/147.04385	
37	9.84	Feruloylquinic acid	369.11801	C_17_H_21_O_9_^+^	0.00	177.05449, 145.02818	
38	10.79	Dillapional-*O*-dihexoside	559.16492	C_24_H_31_O_15_^−^	−1.48	397.11212, 235.06065	
39	11.07	Dimethoxyfuranohydrocoumaroyl-*O*-dihexoside *	589.17474	C_25_H_33_O_16_^−^	1.12	427.12338, 265.07101	
40	15.12	Octyl methoxycinnamate	291.19534	C_18_H_27_O_3_^+^	−0.45	273.18475	+
*Aliphatic acids and Phenolic acids/glycosides*							
41	2.48	Elenolic acid	241.07	C_11_H_13_O_6_^−^	0.72	153.01907, 109.02943	+
42	3.09	Hydroxyisophthalic acid	181.01372	C_8_H_5_O_5_^−^	−3.15	137.02428	
43	3.68	Monotropeoside	445.1333	C_19_H_25_O_12_^−^	−1.62	427.1212,385.1129, 269.1021	
44	3.93	Isopropylmalic acid	175.06	C_7_H_11_O_5_^−^	3.31	157.05049, 115.04002, 113.06070	+
45	4.38	2-Hydroxyisocaproic acid hexoside	293.12308	C_12_H_21_O_8_^−^	−0.05	131.07114	+
46	5.31	Vanilloloside	317.12027	C_14_H_21_O_8_^+^	−8.91	299.10376, 203.05258, 185.04211,	
47	9.70	Syringoylquinic acid	371.09723	C_16_H_19_O_10_^−^	−0.12	249.06111	
48	11.09	Hydroxypropofol O-glucuronide	369.15372	C_18_H_25_O_8_^−^	−1.83	351.14371, 311.11267	
49	11.10	Ptelatoside A *	413.1438	C_19_H_25_O_10_^−^	0.23	351.14352, 311.11240, 269.10214	
50	11.78	Azelaic acid	187.09712	C_9_H_15_O_4_^−^	3.39	125.09694	+
51	11.82	Ptelatoside B	427.15964	C_20_H_27_O_10_^−^	−0.55	325.12830, 161.04520	
52	12.73	Dihydrogalloyl-*O*-hexoside *	329.19583	C_17_H_29_O_6_^−^	−0.11	161.04507	+
*Iridoids*							
53	1.34	Adenosmoside	363.16559	C_16_H_27_O_9_^−^	−1.74	241.00191	+
54	2.61	Geniposidic acid	375.12534	C_16_H_23_O_10_^+^	−8.62	313.12543, 231.08340	
55	3.08	Acetylloganic acid	419.15076	C_18_H_27_O_11_^+^	−9.61	357.15198, 275.11020	
56	3.83	Tudoside	417.10211	C_17_H_21_O_12_^−^	−1.54	285.06100, 241.07098, 152.01129	
57	6.36	Geniposide *	389.14102	C_17_H_25_O_10_^+^	−8.23	327.14093, 245.09958,	
58	8.50	Sweroside	357.11835	C_16_H_21_O_9_^−^	0.96	193.05029	
59	16.01	Valdiate *	311.18552	C_17_H_27_O_5_^+^	0.71	293.17484, 255.12274, 237.11218	+
*Terpenes*							
60	5.89	Euonyminol	365.14465	C_15_H_25_O_10_^−^	−1.49	303.14508, 263.11276, 221.10072	
61	6.64	Phyllaemblic acid B	349.15143	C_15_H_25_O_9_^+^	−6.08	331.12054, 193.12245, 127.03888	
62	7.46	Anisatin	327.10745	C_15_H_19_O_8_^−^	0.02	165.05574, 147.04503	+
63	9.31	Citroside A	385.18555	C_19_H_29_O_8_^−^	−0.38	249.1127, 205.1230, 179.0558	
64	9.75	Unknown diterpene acetate	371.22006	C_23_H_31_O_4_^+^	−4.38	327.20108, 283.17490, 221.13826, 177.11200, 133.08582	
65	9.98	Unknown triterpene	553.37134	C_31_H_53_O_8_^+^	−3.89	535.35925, 351.23840, 267.13351	
66	10.02	Corchoionoside B	399.16492	C_19_H_27_O_9_^−^	−0.10	381.15445, 341.12338, 299.11298	
67	11.70	Diacetoxyl epoxy-apotirucallenetetraol	589.37207	C_34_H_53_O_8_^+^	−2.42	513.32635	
68	12.45	Fruticoside E	645.40393	C_37_H_57_O_9_^−^	−2.28	569.35181, 511.31049	
69	13.22	Unknown terpene	469.33075	C_30_H_45_O_4_^+^	−1.04	451.31940	
70	13.34	Tragopogonsaponin A	647.37665	C_36_H_55_O_10_^−^	−3.59	485.32437,	
71	13.36	Quillaic acid	487.34097	C_30_H_47_O_5_^+^	−1.71	469.33044, 451.31979	
72	13.83	Unknown terpene	427.26733	C_23_H_39_O_7_^+^	−3.98	409.25574, 269.13644	
73	15.36	Amaranthussaponin	955.48846	C_48_H_75_O_19_^−^	−1.30	731.43488, 523.37750, 453.33609	
74	16.43	Momordicinin	439.35724	C_30_H_47_O_2_^+^	0.42	421.34732, 393.35141	
75	16.72	Auraptene	297.15207	C_19_H_21_O_3_^−^	−7.82	240.08189, 183.01169	
76	17.42	Bacobitacin B	599.31854	C_34_H_47_O_9_^+^	−4.87	563.29681, 581.30725, 337.27313	
77	17.76	Unknown terpene *	573.30267	C_32_H_45_O_9_^−^	1.50	409.23471, 391.22437, 317.06357, 243.02704	
78	17.79	Tschimganin	305.17484	C_18_H_25_O_4_^+^	0.34	273.14832, 241.12218	
79	17.86	Unknown terpene	573.30249	C_32_H_45_O_9_^−^	−5.79	409.23468, 391.22430, 317.06351	
80	17.91	Unknown terpene	555.28174	C_28_H_43_O_11_^−^	3.15	299.04340, 225.00691	+
81	18.20	Acetyloxy torilolone	293.1786	C_17_H_25_O_4_^−^	3.35	96.95988	
82	19.10	Unknown terpene	409.23511	C_26_H_33_O_4_^−^	−5.44	152.99554	
83	23.83	Yonogenin	433.32993	C_27_H_45_O_4_^+^	−3.02	307.19000, 293.17441, 149.02310	
84	26.62	Acetoxy hydroxymethoxy-oleanene	515.41339	C_33_H_55_O_4_^+^	7.57	329.21429	
*Fatty acids*							
85	9.05	Traumatic acid	229.14305	C_12_H_21_O_4_^+^	−1.68	211.13272, 193.12216	
86	14.11	Trihydroxyoctadecenoic acid	329.2326	C_18_H_33_O_5_^−^	1.06	311.22211, 229.14413, 171.10242	
87	14.19	Oxo octadecadienonic acid	295.22662	C_18_H_31_O_3_^+^	−0.51	277.21600	+
88	15.19	Octadecenedioic acid	313.23642	C_18_H_33_O_4_^−^	−2.92	295.22638, 277.21603	
89	17.06	Octadecatrienoic acid	311.2218	C_18_H_31_O_4_^−^	0.37	-	
90	17.98	Hydroxyoctadecadienoic (Coriolic acid)	295.22693	C_18_H_31_O_3_^−^	0.54	277.21640, 195.13849, 179.14368	+
91	18.85	Stearyl citrate	443.29956	C_24_H_43_O_7_^−^	−1.74	279.23203	
92	19.46	Palmitoylhexitol	419.29932	C_22_H_43_O_7_^−^	−2.41	255.23193	
93	19.80	Methyl Linolenate	293.24759	C_19_H_33_O_2_^+^	0.28	261.22107, 243.21054	+
94	22.71	Methyl linoleate	295.26309	C_19_H_35_O_2_^+^	−0.23	263.23694, 245.22644	
95	24.95	Glyceryl ricinolpalmitein	607.4917	C_37_H_67_O_6_^+^	−2.50	589.48108	
*Others*							
96	1.69	Mannitol	181.0715	C_6_H_13_O_6_^−^	4.72	163.0610, 101.02435	
97	1.77	Homovanillyl-*O*-hexoside	343.10147	C_15_H_19_O_9_^−^	1.32	181.05017, 163.03961	
98	2.28	Uralenneoside	285.06085	C_12_H_13_O_8_^−^	1.25	153.01913	
99	5.39	Celephthalide derivative	405.21118	C_19_H_33_O_9_^−^	−1.43	405.21118	
100	5.70	Acutilactone	409.32681	C_25_H_45_O_4_^+^	−10.81	276.21655, 160.13304	
101	5.87	Benzyl-*O*-hexoside	269.10275	C_13_H_17_O_6_^−^	−2.92	171.47818	
102	8.90	Benzyl-*O*-hexosyl pentoside	401.14337	C_18_H_25_O_10_^−^	−2.13	269.10239, 161.04523	
103	9.00	Dactylorhin C	351.12897	C_14_H_23_O_10_^−^	−1.13	333.11838, 267.07202, 249.06105	
104	9.19	Dihomo-jasmonic acid	237.14897	C_14_H_21_O_3_^−^	1.89	171.11749	
105	9.67	Glehlinoside C	551.17535	C_26_H_31_O_13_^−^	−1.03	389.12271, 193.05025	
106	9.98	Falcarindiol	261.18484	C_17_H_25_O_2_^+^	−0.26	219.17432, 205.12210	
107	10.07	Citrusin A *	537.19586	C_26_H_33_O_12_^−^	−1.48	489.17389, 327.12231	
108	10.41	Hydroxydihydrojasmonic acid-*O*-hexoside	435.18478	C_19_H_31_O_11_^−^	−3.01	389.18011	
109	11.27	Celephthalide C	371.1694	C_18_H_27_O_8_^−^	−1.74	354.15955	
110	12.10	Hydroxymethyl Celephthalide C	403.19452	C_19_H_31_O_9_^−^	−4.31	287.22067,	
111	12.90	Unknown phthalate derivative	353.19312	C_19_H_29_O_6_^+^	−7.77	235.13074, 203.05276	
112	13.84	Pterosin P	235.13051	C_14_H_19_O_3_^+^	−10.04	217.15845, 179.10645	
113	17.17	Unknown	595.28656	C_27_H_47_O_14_^−^/ C_34_H_43_O_9_^−^	−1.5/ 3.9	415.22418, 279.23230, 241.01138	
114	17.97	Unknown *	325.18344	C_14_H_29_O_8_^−^	−6.93	183.01180	
115	18.39	Benthamianone *	433.23505	C_28_H_33_O_4_^−^	−5.28	152.99571	

*: Major metabolites detected in TAE of celery aerial parts by UPLC/ESI/TOF-MS analysis, +: major metabolites detected in DCM of celery aerial parts by UPLC/ESI/TOF-MS analysis.

## Data Availability

Data are available upon request from the corresponding author (A.M.E.).

## Supplementary Materials

The following are available online, Figure S1: ESI-MS/MS Spectrum of peak (5) in the positive ion mode, Figure S2: ESI-MS/MS Spectrum of peak (6) in the negative ion mode, Figure S3: ESI-MS/MS Spectrum of peak (7) in the positive ion mode, Figure S4: ESI-MS/MS Spectrum of peak (3) in the positive ion mode, Figure S5: ESI-MS/MS Spectrum of peak (4) in the positive ion mode, Figure S6: ESI-MS/MS Spectrum of peak (9) in the positive ion mode, Figure S7: ESI-MS/MS Spectrum of peak (10) in the positive ion mode, Figure S8: ESI-MS/MS Spectrum of peak (32) in the positive ion mode, Figure S9: ESI-MS/MS Spectrum of peak (21) in the positive ion mode, Figure S10: ESI-MS/MS Spectrum of peak (22) in the negative ion mode, Figure S11: ESI-MS/MS Spectrum of peak (28) in the positive ion mode, Figure S12: ESI-MS/MS Spectrum of peak (25) in the positive ion mode, Figure S13: ESI-MS/MS Spectrum of peak (34) in the negative ion mode, Figure S14: ESI-MS/MS Spectrum of peak (36) in the negative ion mode, Figure S15: ESI-MS/MS Spectrum of peak (37) in the positive ion mode, Figure S16: ESI-MS/MS Spectrum of peak (63) in the negative ion mode, Figure S17: ESI-MS/MS Spectrum of peak (75) in the negative ion mode, Figure S18: ESI-MS/MS Spectrum of peak (78) in the positive ion mode.

## Sample Availability

Samplesare available from the authors.
