# Supplementary material for: Antioxidant, Antimicrobial Activities and Characterization of Polyphenol-Enriched Extract of Egyptian Celery (Apium graveolens L., Apiaceae) Aerial Parts via UPLC/ESI/TOF-MS
Source: Molecules. 2022 Jan 21;27(3):698. doi: 10.3390/molecules27030698 (PMC8838201; doi:10.3390/molecules27030698)
Supplement: Supplementary file 1 [file molecules-27-00698-s001.zip › molecules-1552787-supplementary.pdf]

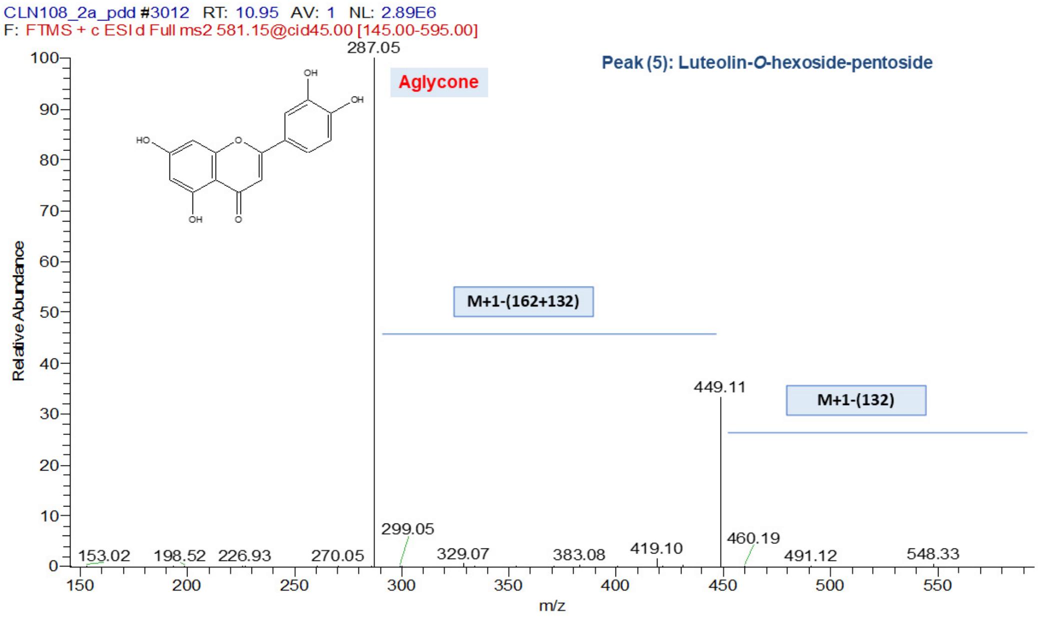

Figure S1: ESI-MS/MS Spectrum of peak (5) in the positive ion mode

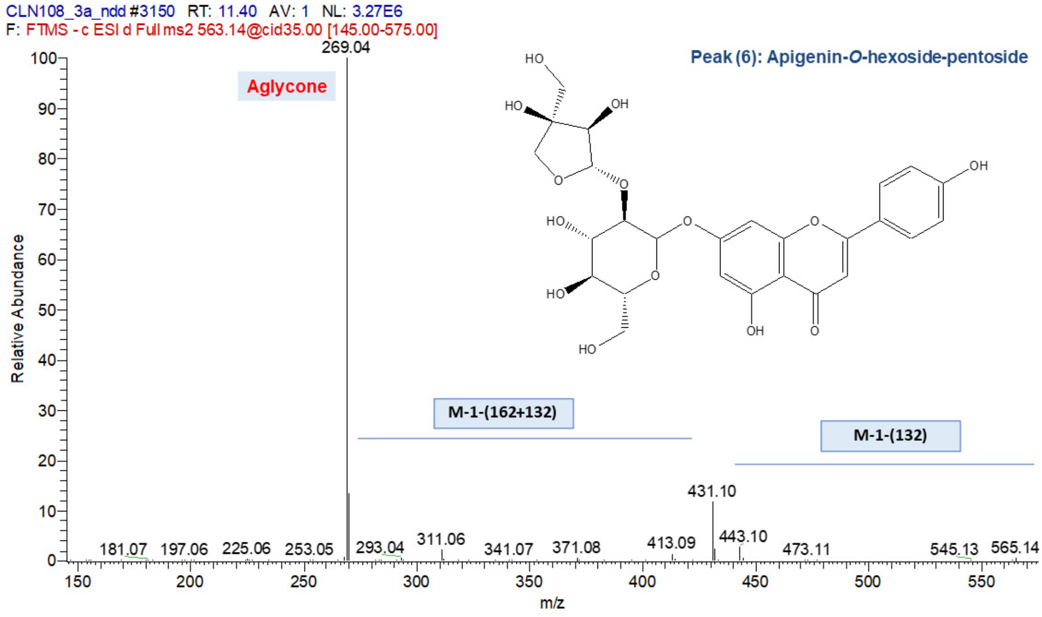

Figure S2: ESI-MS/MS Spectrum of peak (6) in the negative ion mode

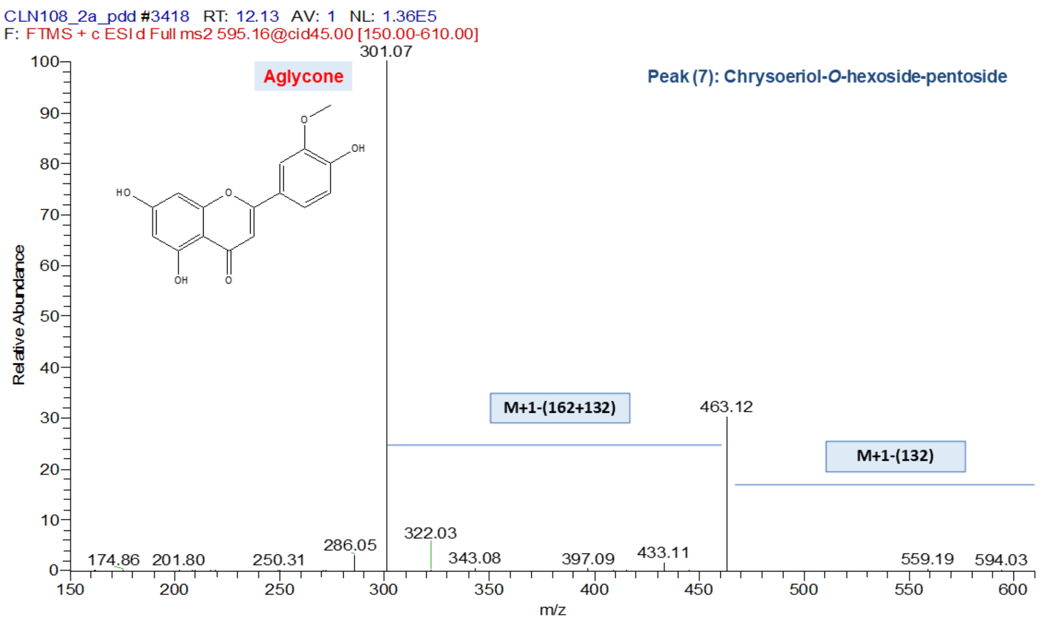

Figure S3: ESI-MS/MS Spectrum of peak (7) in the positive ion mode

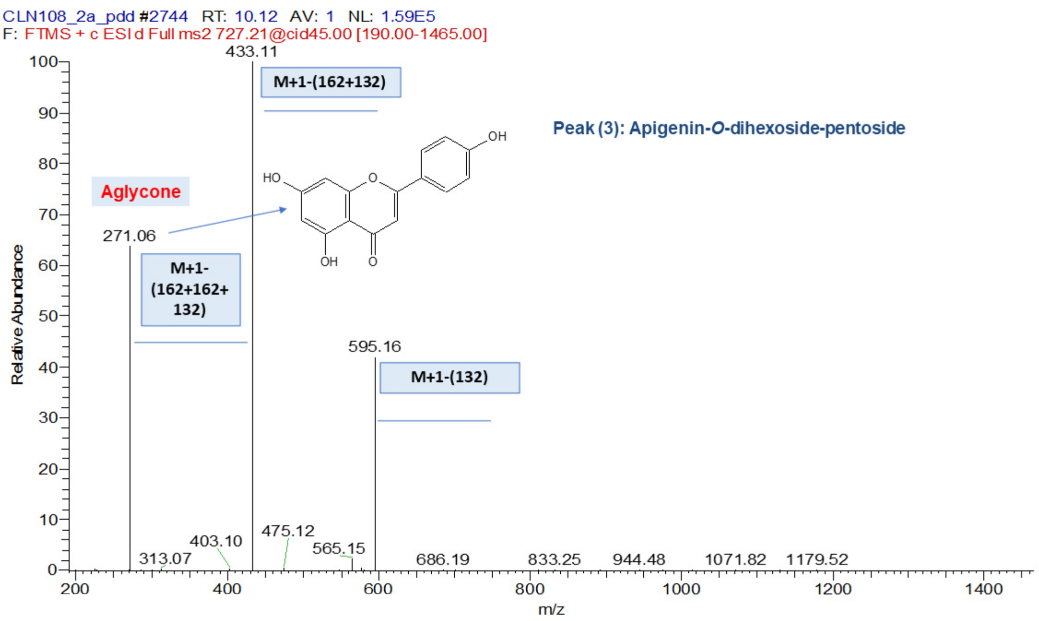

Figure S4: ESI-MS/MS Spectrum of peak (3) in the positive ion mode

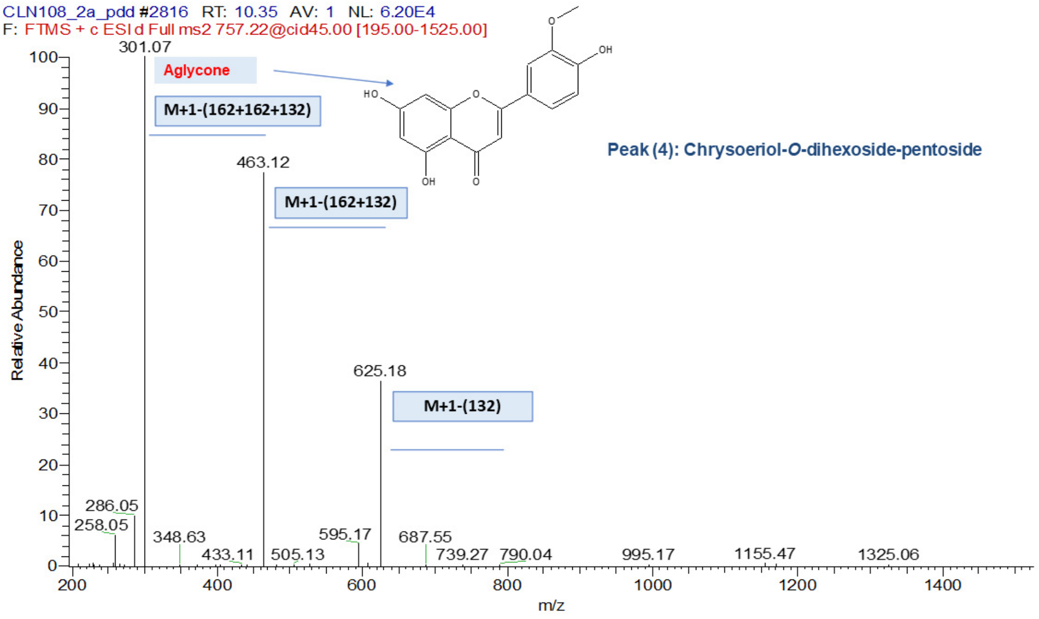

ESI-MS/MS Spectrum of peak (4) in the positive ion mode

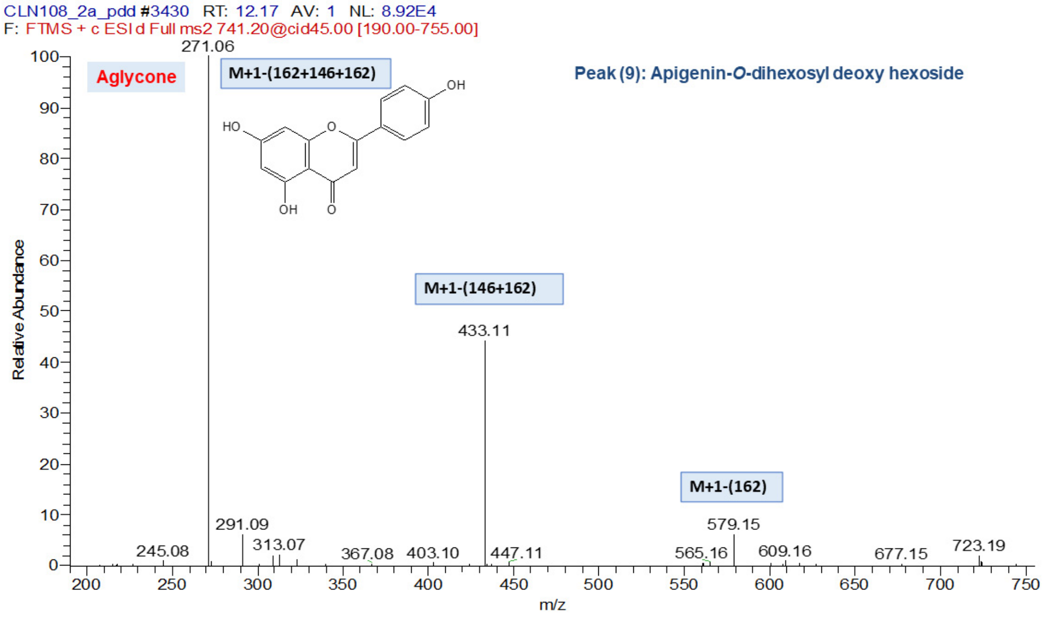

Figure S6: ESI-MS/MS Spectrum of peak (9) in the positive ion mode

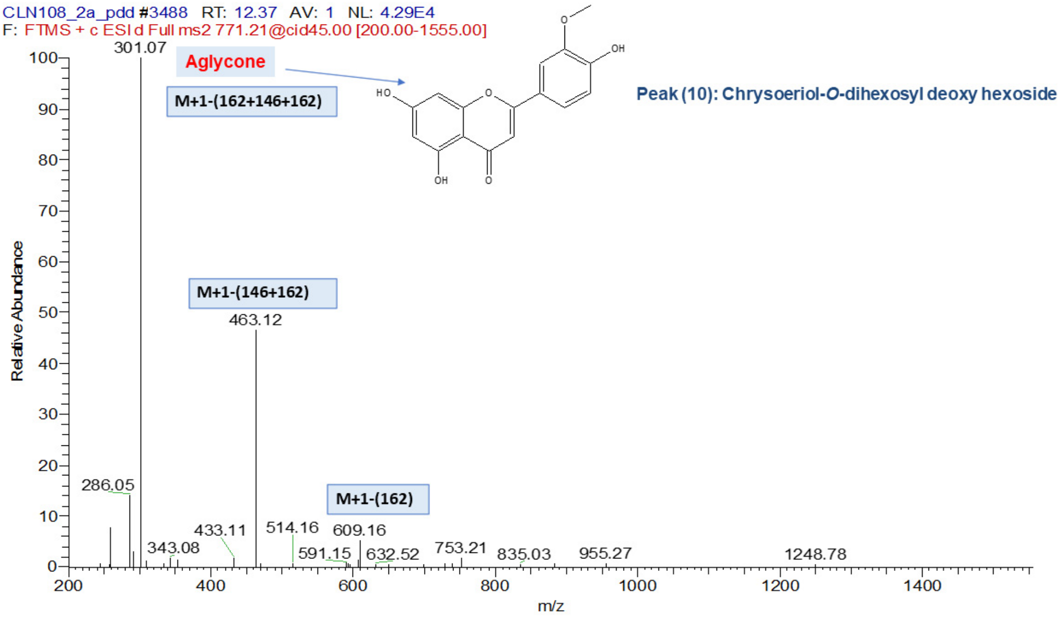

Figure S7: ESI-MS/MS Spectrum of peak (10) in the positive ion mode

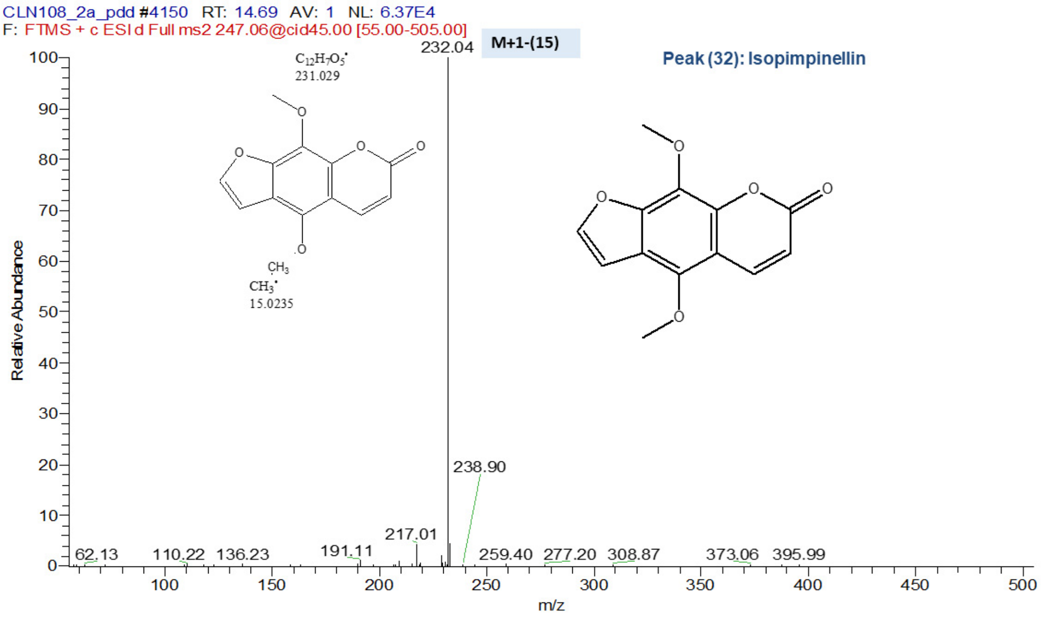

Figure S8: ESI-MS/MS Spectrum of peak (32) in the positive ion mode

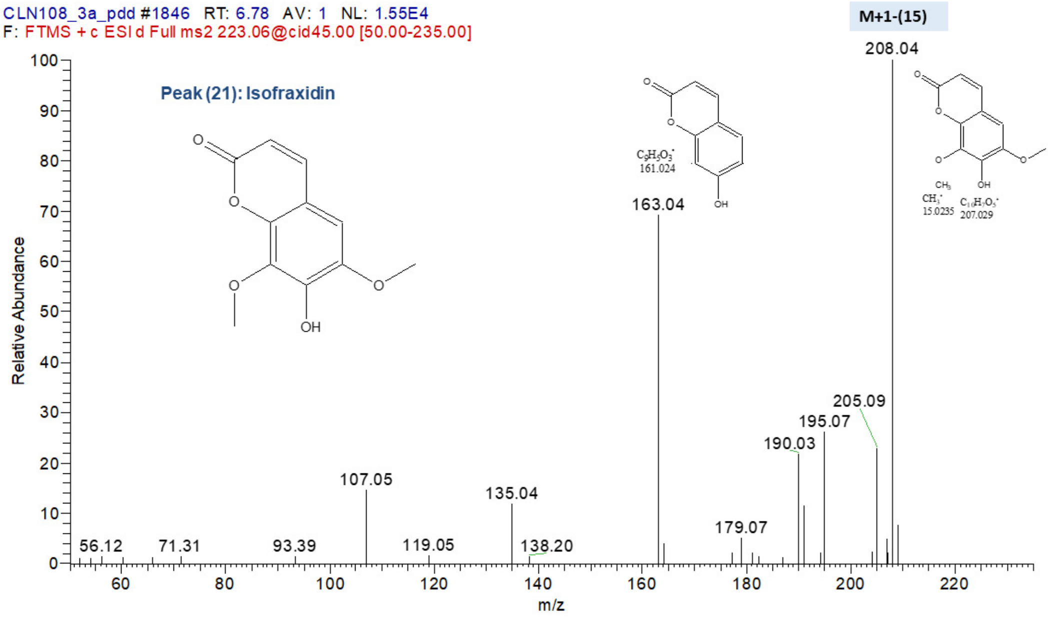

Figure S9: ESI-MS/MS Spectrum of peak (21) in the positive ion mode

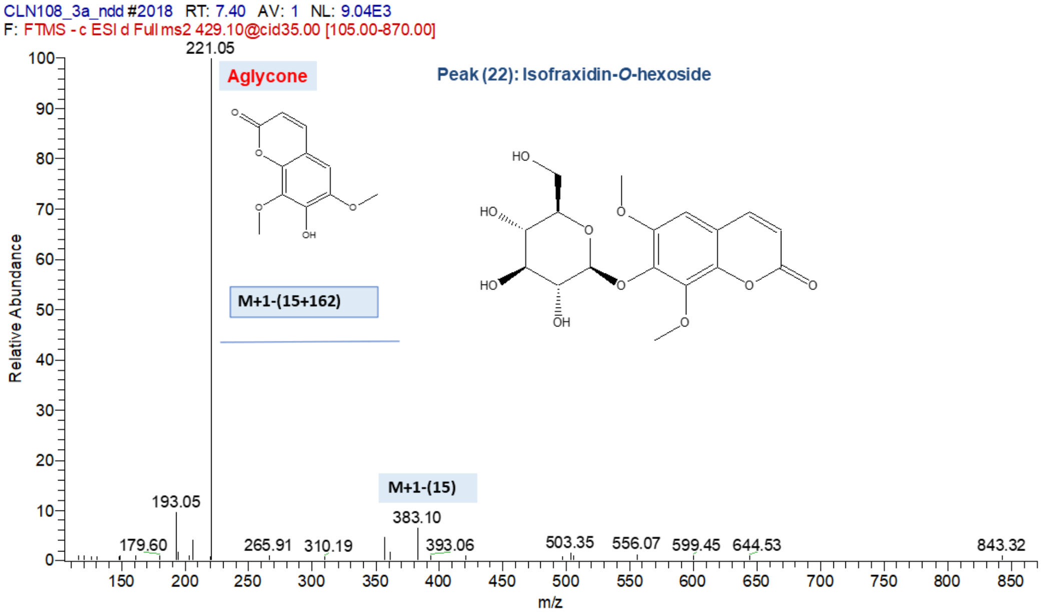

Figure S10: ESI-MS/MS Spectrum of peak (22) in the negative ion mode

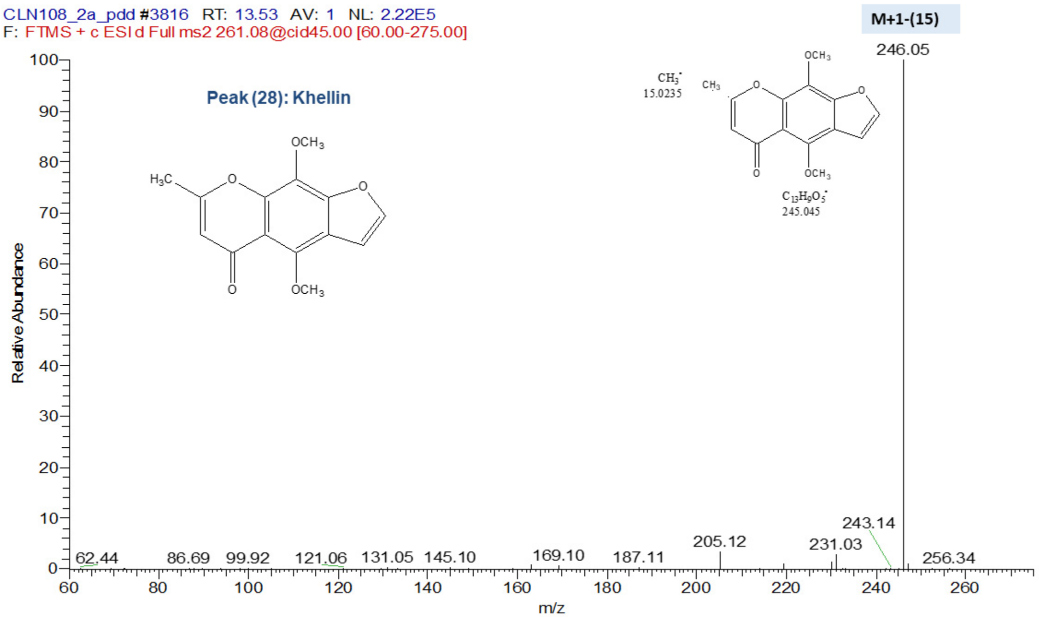

Figure S11: ESI-MS/MS Spectrum of peak (28) in the positive ion mode

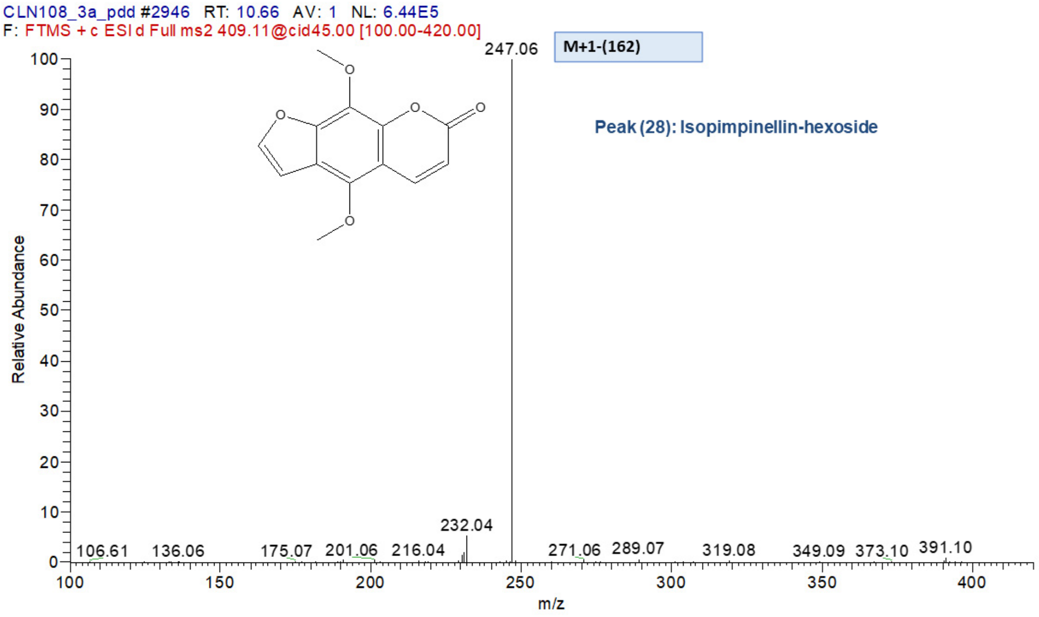

Figure S12: ESI-MS/MS Spectrum of peak (25) in the positive ion mode

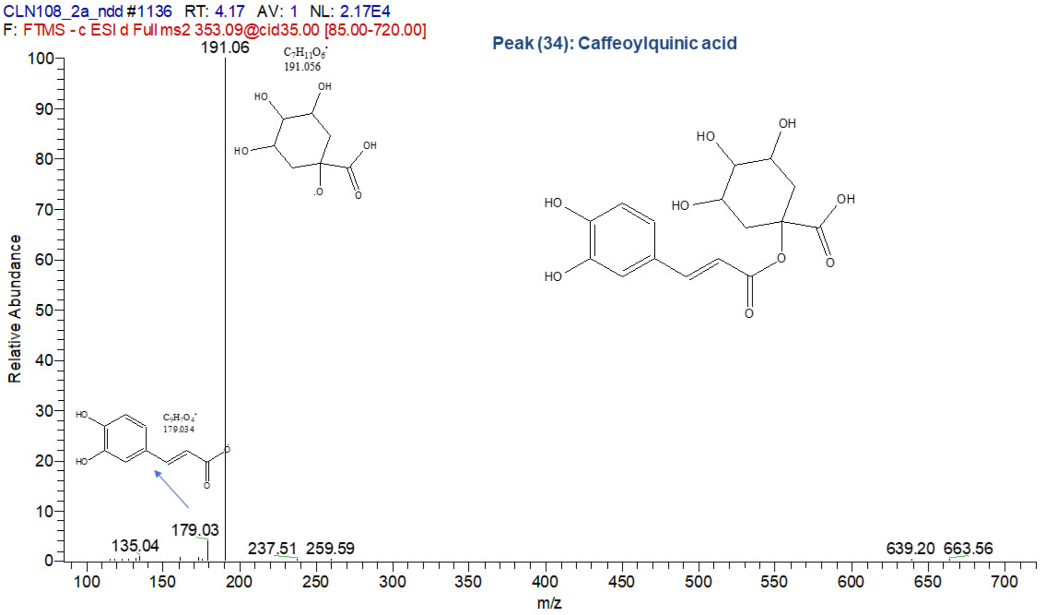

Figure S13: ESI-MS/MS Spectrum of peak (34) in the negative ion mode

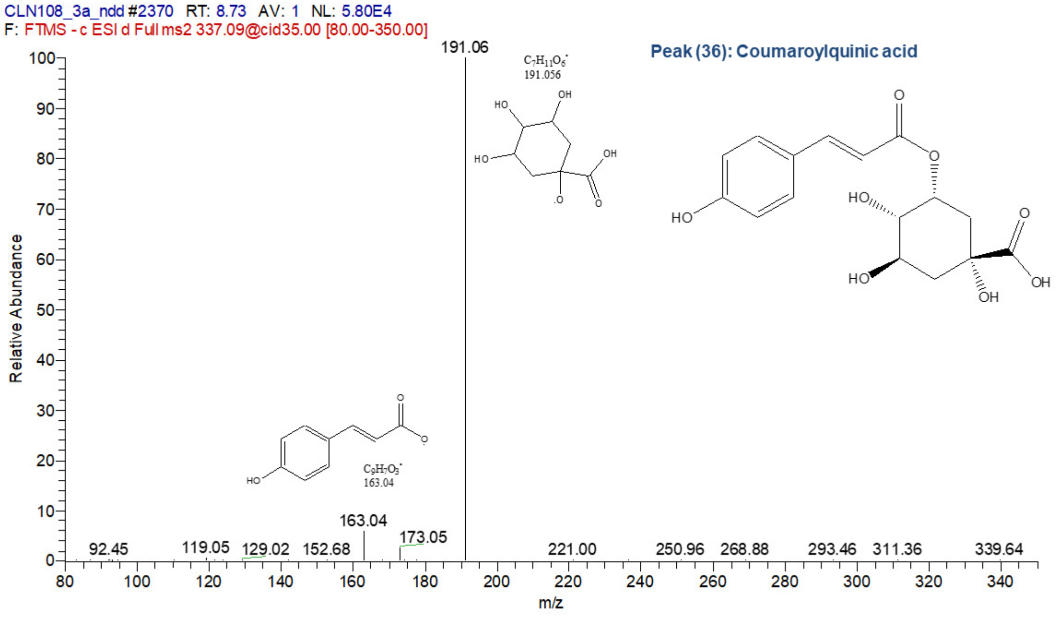

Figure S14: ESI-MS/MS Spectrum of peak (36) in the negative ion mode

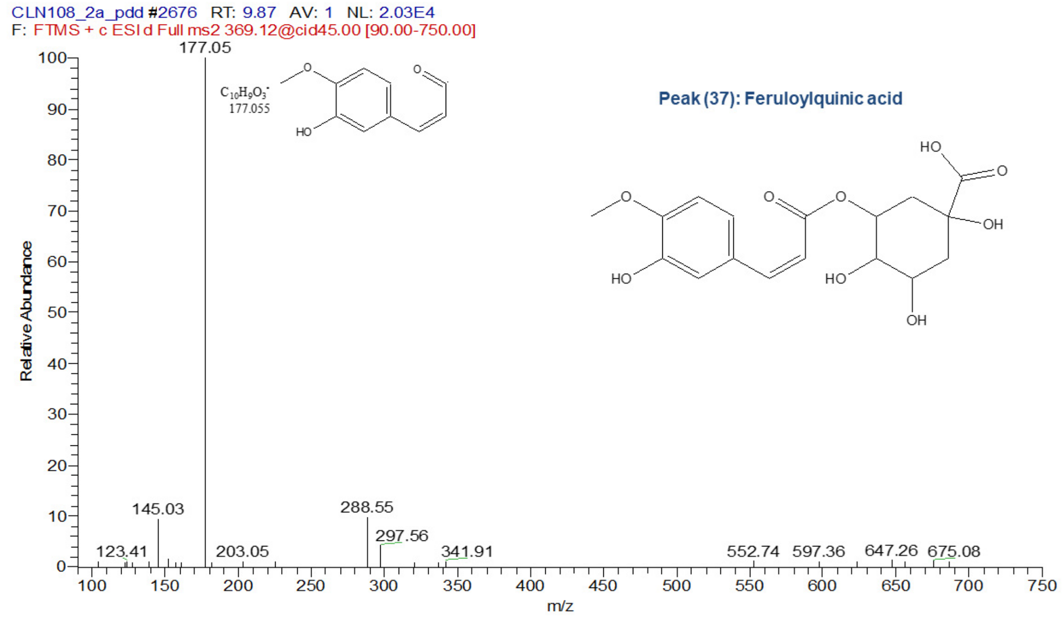

Figure S15: ESI-MS/MS Spectrum of peak (37) in the positive ion mode

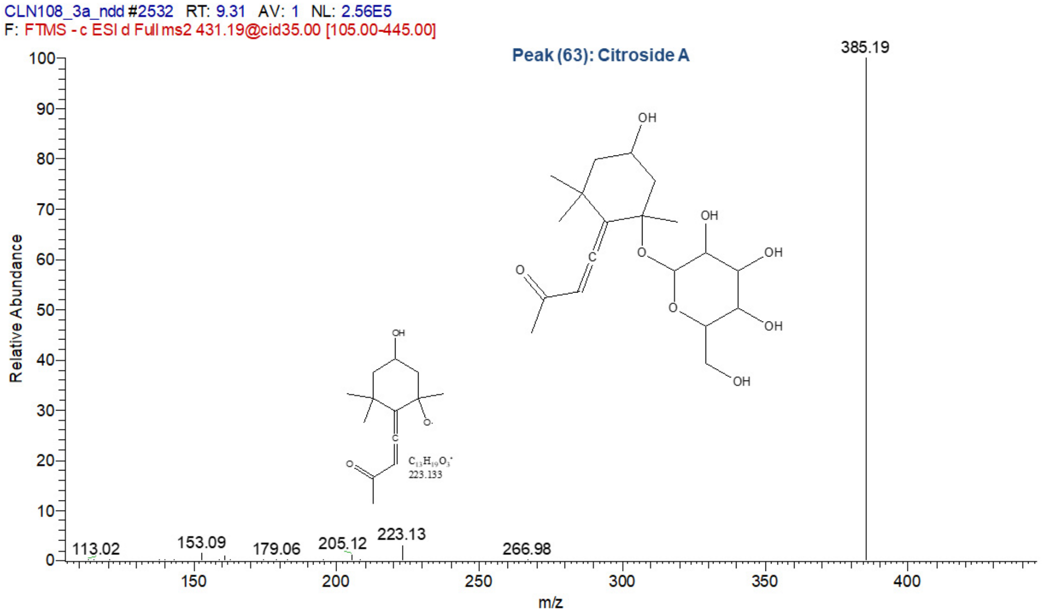

Figure S16: ESI-MS/MS Spectrum of peak (63) in the negative ion mode

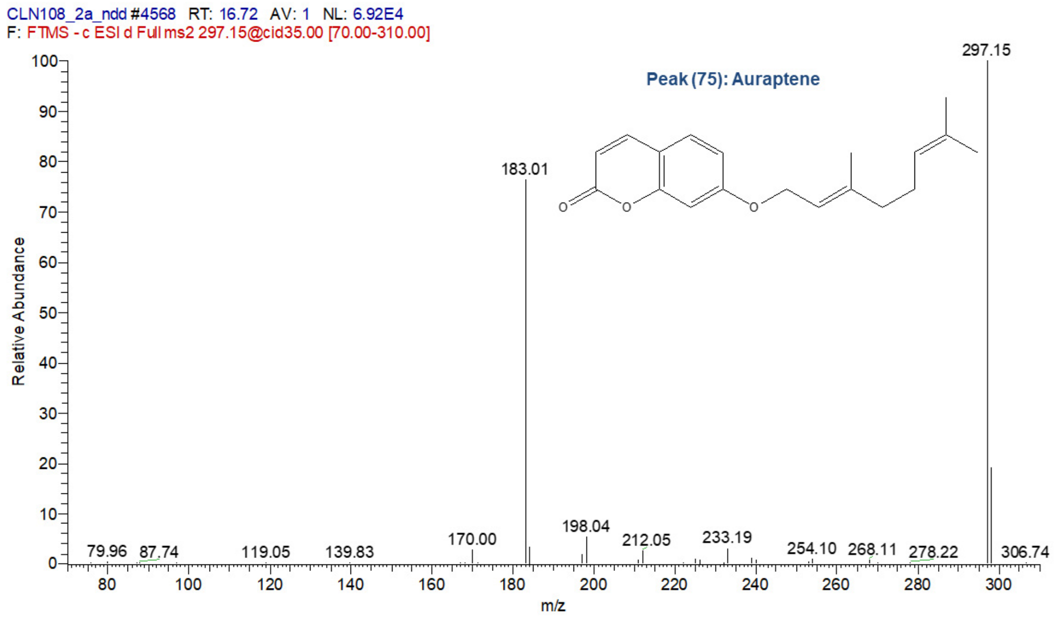

Figure S17: ESI-MS/MS Spectrum of peak (75) in the negative ion mode

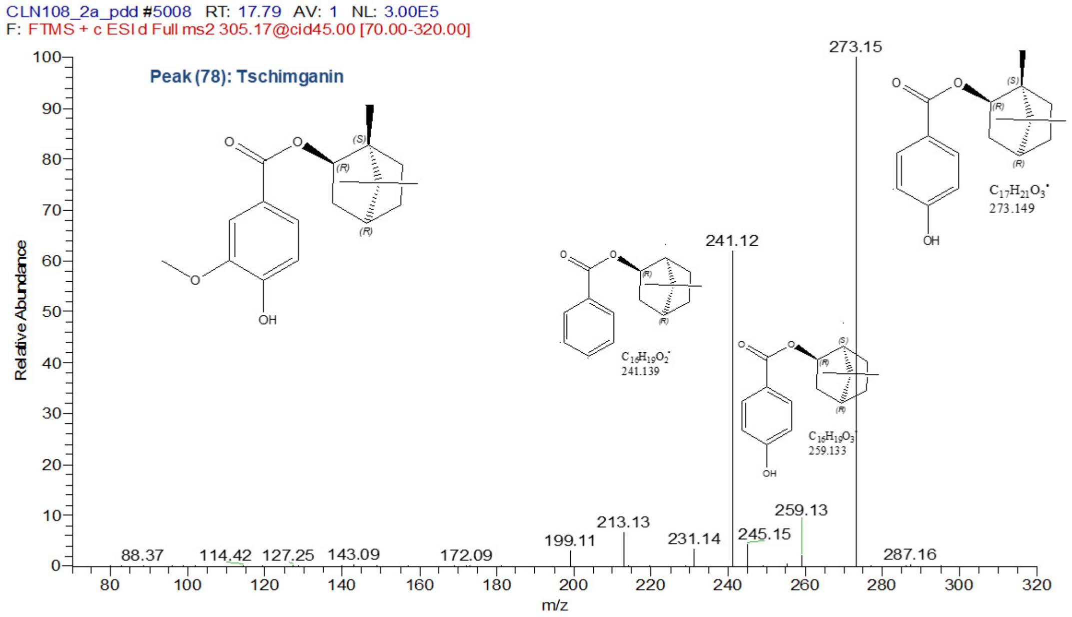

Figure S18: ESI-MS/MS Spectrum of peak (78) in the positive ion mode
